# Identification and Distribution of Pathogens in a Major Tertiary Hospital of Indonesia

**DOI:** 10.3389/fpubh.2019.00395

**Published:** 2020-01-31

**Authors:** Nyoman Sri Budayanti, Dewi N. Aisyah, Nengah N. D. Fatmawati, Ni M. A. Tarini, Zisis Kozlakidis, Wiku Adisasmito

**Affiliations:** ^1^Microbiology Clinic Department, Faculty of Medicine, Udayana University, Denpasar, Indonesia; ^2^Microbiology Clinic Department, Sanglah General Hospital, Denpasar, Indonesia; ^3^Institute of Health Informatics, University College London, London, United Kingdom; ^4^Faculty of Public Health, Universitas Indonesia, Jakarta, Indonesia; ^5^International Agency for Research on Cancer, World Health Organization, Lyon, France

**Keywords:** pathogen distribution, drug resistance, Indonesia, tertiary hospital, multi-drug-resistance organisms

## Abstract

The nosocomial persistence of multiple drug resistance organisms constitutes a global threat. Healthcare-setting acquired infections are subject to substantial selection pressure and are frequently associated with drug resistance. As part of the microbiological surveillance of the Sanglah tertiary referral hospital in the island province of Bali, the distribution of bacterial pathogen and their relative susceptibilities were recorded over a 30 months period. This is the first such detailed study benchmarking the type and sensitivity of bacterial pathogens in a major tertiary referral hospital within Indonesia and it is hoped that it will lead to similar reports in the near future, while informing local and national antimicrobial stewardship policies.

## Introduction

Multi-drug-resistance organisms (MDROs) are a global threat that have stemmed—at least in part—from poor antimicrobial stewardship (1, 2). Infection with an MDRO may complicate clinical treatment and be associated with poorer outcomes including increased length of hospital stay and mortality (3–5). MDR is defined as acquired non-susceptibility to at least one agent in three or more antimicrobial categories (6). Infections acquired in healthcare settings are subject to substantial selection-pressure (for example reflecting high rates of antibiotic usage) and are frequently associated with drug resistance: effective empirical treatment is therefore of particular importance (7, 8). Antimicrobial stewardship promotes the selection of the empirical therapy that is the most appropriate given the knowledge of local patterns of drug resistance and also advocates switching to a narrower spectrum therapy upon confirmation of the causative organism and its susceptibility profile. Accurate and timely information on local and national patterns of organisms causing infection and their drug-resistance profile is therefore paramount for good clinical management and benchmarking (9, 10).

According to the Basic Health Research (Riset Kesehatan Dasar) 2013, a national health survey which was conducted across 33 provinces in Indonesia with a total of 1,027,763 subject populations, the prevalence of upper respiration tract infection, and pneumonia were 25 and 4.5%, respectively (11). However, the prevalence of Hospital Acquired Infection (HAI) in Indonesia was not available on a national level. Similarly, the information of HAI in low and middle income countries was very limited. WHO reported only 23 of 147 (15.6%) developing countries provided national data on health care associated infection with pooled prevalence of 10.1% in 2010 (12). As for Indonesia, a single study found reported HAI prevalence of an overall 7.1% in two teaching hospitals in East Java (13). To date, no studies have reported the pattern of bacteria causing clinical infection and the associated drug-resistance profile for Indonesia.

Sanglah Hospital is a tertiary referral center and major healthcare hub for East Indonesia. The hospital is situated on the island province of Bali, an international tourist destination, which attracts more than 9 million tourists a year. In 2015 the annual number of visiting tourists outnumbered the resident population of Bali by a factor of two (14). Sanglah hospital therefore hosts a clinical population that is derived from geographically dispersed locations across Indonesia and the world, though for hospitalization purposes local patients tend to still form the greater majority and this also holds true for the majority of samples analyses reported in the current manuscript. This diverse and highly mobile patient group has the potential to facilitate the spread of MDROs between both healthcare staff and patients and may make the selection of appropriate empirical therapy even more complex.

The aim of this study is to describe the relative distribution and sensitivity of bacteria causing infections in patients attending the Sanglah hospital. This knowledge will help guide the selection of appropriate empirical treatments at a local level and will provide a benchmark for comparison at other sites in Indonesia and Asia. To the best of our knowledge, this study is the first to report the type and sensitivity of bacteria in a major Indonesian tertiary referral hospital.

## Materials and Methods

Data on the type of bacteria and their sensitivity to antibiotics that are routinely used in Sanglah Hospital were retrospectively identified from clinical samples taken from patients between July 1st 2013 and December 30th 2015. The data were inclusive of all patient results within the hospital Microbiology unit, irrespective of any patient-specific parameters, such as age groups, hospital admission patterns or pre-existing patient co- morbidities, in order to benchmark bacterial presence and MDROs at a population level. Data were collected from culture-data records in the Microbiology department in Sanglah Hospital.

All bacteria with microbiology results consistent with clinical infection (culture result was consistent with Gram stain result; particularly in conjunction with raised white blood cell counts in the Gram stain) were reported. However, bacteria that were considered likely to be a contaminant (for example those reflecting normal flora according to Garcia (15), or where sensitivity results were not available, were excluded. Bacteria were isolated from clinical specimens including blood, urine, sputum, cerebrospinal fluid (CSF), and other specimens (vitreous fluid, pleural fluid, synovial fluid, throat swab, feces). Specimens were collected and processed following the Sanglah Microbiology laboratory standard operational procedure (SOP) for culture which is based on the Clinical Microbiology Procedure Handbook by Garcia (15). Identification and antibiotic sensitivity tests were conducted based on the micro- dilution method using a Vitek-2 Compact machine following the exact recommendations of the 2015 Clinical and Laboratory Standards Institute (CLSI). Cefoxitin non-susceptibility was used as a marker of methicillin resistance in *Staphylococcus aureus* (MRSA).

Extended-Spectrum Beta-Lactamase-Producing *Escherichia coli* Isolates (ESBL) screening was initially performed with the CLSI confirmatory test, using both cefotaxime (30 mg) and ceftazidime (CAZ) (30 mg) disks alone and in combination with clavulanic acid (CA) (10 mg) (Eiken Chemical, Tokyo, Japan). The test was considered positive when the diameter of the growth-inhibitory zone around either the cefotoxamine or the CAZ disk in combination with CA increased by ≥5 mm compared to the growth-inhibitory zone around the disk containing CTX or CAZ alone (16).

## Results

During the 30-months study period a total of 12,286 specimens were tested. This period is coincidental with the national reporting structures and the most recently available and audited results were used. Urine (*n* = 3,849, 31.32%) and blood (*n* = 2,942, 23.94%) were the most common specimen types. Of the 12,286 specimens assessed, 7,392 (60%) showed bacteria growth and a further 320 (3%) were positive for yeast or fungi (*n* = 310, 3%) or normal flora (*n* = 10, <0.1%). Thirty seven percent of specimens showed no growth: the proportion of samples with no growth was highest for blood (*n* = 1,813, 62%) and urine (*n* = 1,220, 32%) (Table 1).

**Table 1:** Microbiology of clinical samples cultured at Sanglah General Hospital July 2013–Dec 2015.

**Organism**	**All specimens *n* (%)**	**Blood *n* (%)**	**Urine *n* (%)**	**Sputum *n* (%)**	**CSF *n* (%)**	**Wound *n* (%)**	Other^*^n (%)
Bacteria	7,392 (60)	1,090 (37)	2,516 (65)	1,508 (84)	183 (24)	1,315 (74)	780 (68)
Yeast/Fungi	310 (3)	39 (1)	111 (3)	137 (8)	6 (1)	5 (0)	12 (1)
Normal flora	10 (0)	0 (0)	2 (0)	2 (0)	0 (0)	0 (0)	6 (1)
No growth	4,574 (37)	1,813 (62)	1,220 (32)	141 (8)	588 (76)	460 (26)	352 (31)
Total	12,286 (100)	2,942 (100)	3,849 (100)	1,788 (100)	777 (100)	1,780 (100)	1,150 (100)

**Figure 1:**
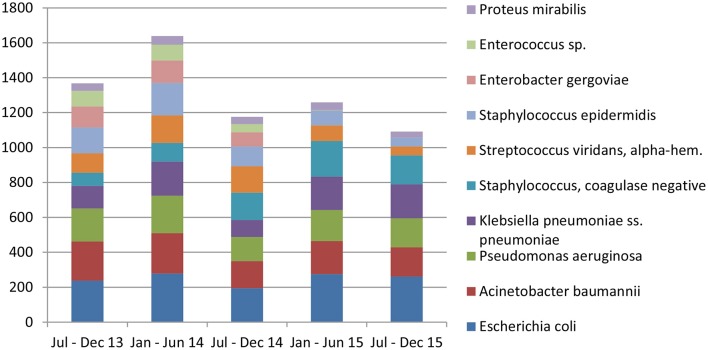
The distribution of top 10 bacteria since July–December 2013 until July–December 2015.

**Figure 2:**
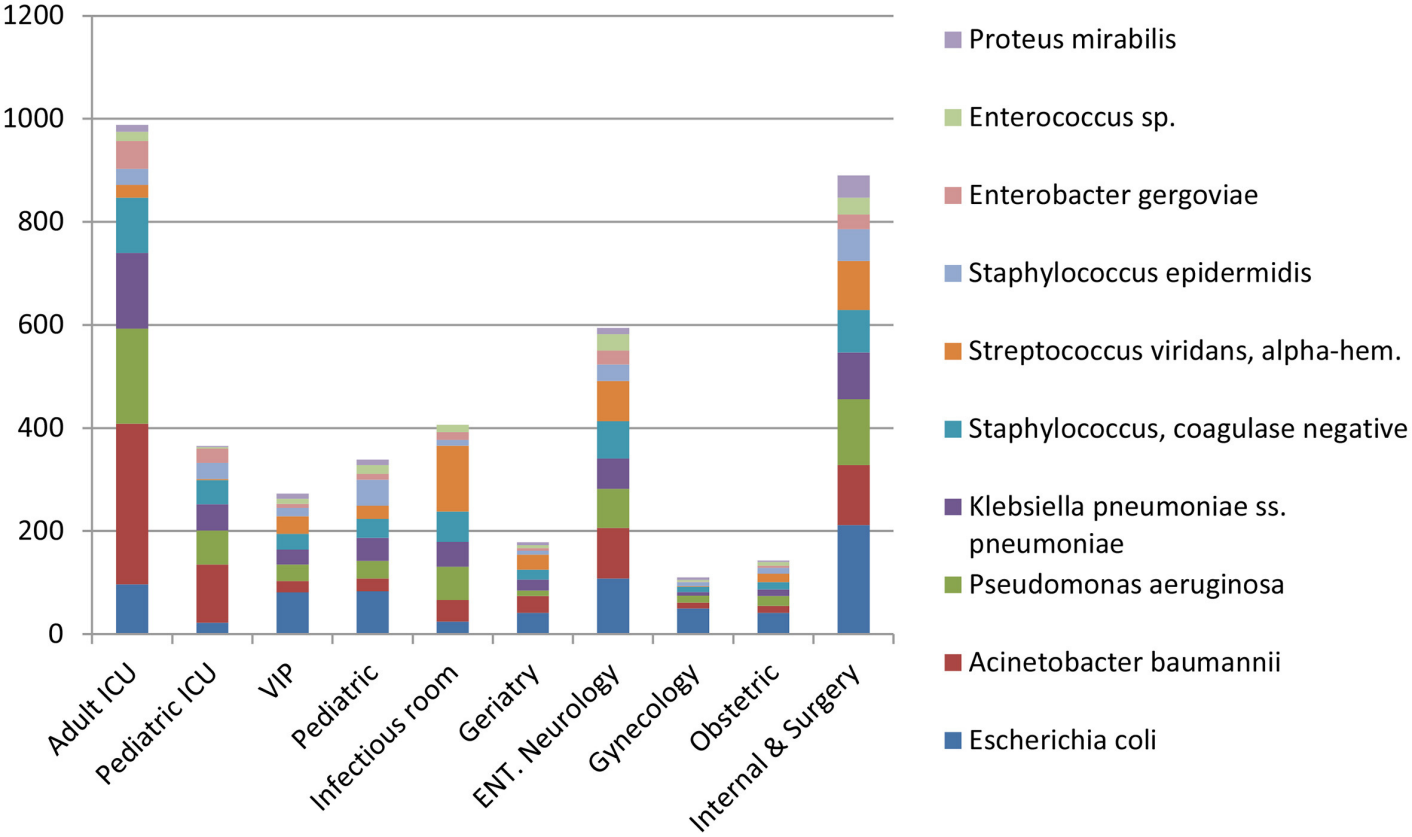
Proportion of bacteria causing infection by ward (%)—top 10.

**Figure 3:**
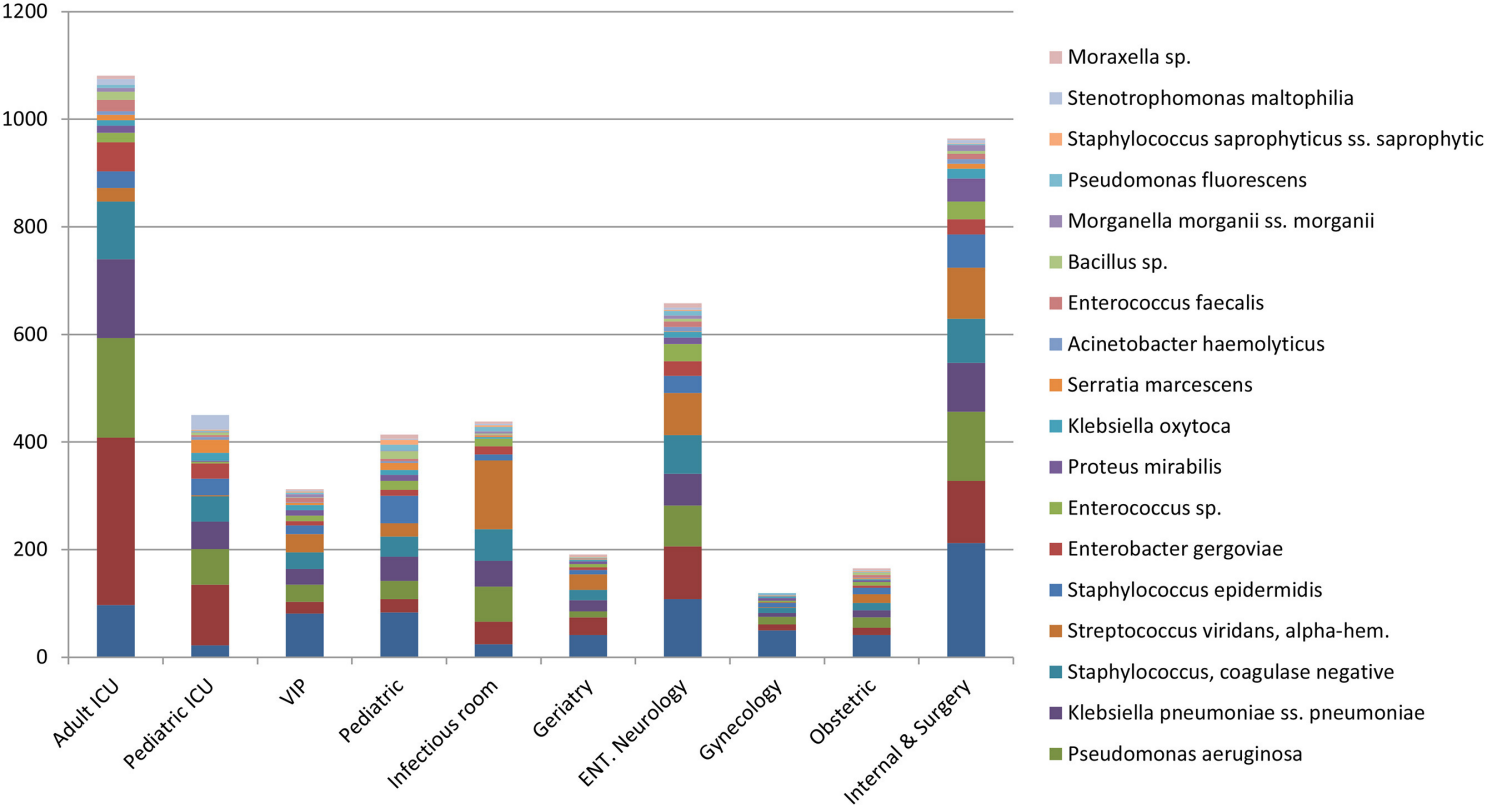
Proportion of bacteria causing infection by ward (%)—top 20.

*Escherichia coli* were the most commonly isolated bacteria (17%), followed by *Acinetobacter baumannii* (13%), *Pseudomonas aeruginosa* (11%), *Klebsiella pneumoniae* (10%), and Coagulase-negative staphylococci (10%). The dominant bacteria differed according to specimen type; for example *E. coli* from urine samples, *Klebsiella pneumoniae* from sputum and *Acinetobacter baumannii* from swabs or other tissue. Coagulase-negative staphylococci were also commonly isolated bacteria (Table 2). The relative occurrence of the isolated bacteria was consistent across the investigated 30 months period, as seen in six-monthly reporting windows (Figure 1). *A. baumannii* was predominantly found in adult and pediatric Intensive Care Units. *E. coli* was most commonly isolated in geriatric, privately serviced hospital rooms, internal medicine and surgical settings. Viridans streptococci and coagulase- negative staphylococci (both part of normal upper respiratory tract flora) were most frequently cultured in infectious disease wards and wards for Human Immunodeficiency Virus(HIV) and Tuberculosis (TB) patients (Figures 2, 3).

**Table 2:** The distribution of bacteria based on type of specimen July 2013–December 2015.

**Bacteria**	**Total *n* (%)**	**Blood *n* (%)**	**CSF *n* (%)**	**Sputum *n* (%)**	**Urine *n* (%)**	**Wound *n* (%)**	**Other *n* (%)**
*Escherichia coli*	1,246 (17)	136 (19)	295 (20)	182 (11)	788 (16)	239 (16)	111 (11)
*Acinetobacter baumannii*	969 (13)	112 (16)	192 (13)	198 (12)	684 (13)	198 (13)	198 (19)
*Pseudomonas aeruginosa*	835 (11)	55 (8)	143 (9)	200 (12)	590 (12)	151 (10)	158 (15)
*Klebsiella pneumoniae ss. pneumoniae*	772 (10)	101 (14)	109 (7)	212 (13)	582 (11)	144 (10)	85 (8)
*Staphylococcus, coagulase negative*	711 (10)	84 (12)	149 (10)	200 (12)	615 (12)	75 (5)	149 (15)
*Streptococcus viridans, alpha-hem*.	560 (8)	61 (8)	136 (9)	121 (7)	440 (9)	140 (9)	42 (4)
*Staphylococcus epidermidis*	584 (8)	26 (4)	186 (12)	163 (10)	389 (8)	117 (8)	59 (6)
*Enterobacter gergoviae*	331 (4)	22 (3)	62 (4)	84 (5)	160 (3)	93 (6)	44 (4)
*Enterococcus sp*.	231 (3)	26 (4)	37 (2)	30 (2)	124 (2)	77 (5)	29 (3)
*Proteus mirabilis*	211 (3)	21 (3)	59 (4)	52 (3)	148 (3)	34 (2)	21 (2)
*Klebsiella oxytoca*	153 (2)	12 (2)	7 (0)	30 (2)	77 (2)	45 (3)	33 (3)
*Serratia marcescens*	137 (2)	10 (1)	16 (1)	34 (2)	78 (2)	30 (2)	21 (2)
*Acinetobacter haemolyticus*	62 (1)	2 (0)	7 (0)	34 (2)	23 (0)	24 (2)	2 (0)
*Enterococcus faecalis*	113 (2)	1 (0)	41 (3)	22 (1)	94 (2)	0 (0)	30 (3)
*Bacillus sp*.	91 (1)	10 (1)	29 (2)	18 (1)	69 (1)	4 (0)	7 (1)
*Morganella morganii ss. morganii*	88 (1)	10 (1)	4 (0)	22 (1)	39 (1)	24 (2)	13 (1)
*Pseudomonas fluorescens*	87 (1)	4 (1)	12 (1)	31 (2)	49 (1)	39 (3)	2 (0)
*Staphylococcus saprophyticus ss. saprophytic*	74 (1)	5 (1)	7 (0)	28 (2)	40 (1)	21 (1)	9 (1)
*Stenotrophomonas maltophilia*	72 (1)	14 (2)	7 (0)	11 (1)	53 (1)	1 (0)	4 (0)
*Moraxella sp*.	65 (1)	7 (1)	14 (1)	22 (1)	37 (1)	25 (2)	9 (1)

Most isolated Gram-positive bacteria were susceptible to linezolid and vancomycin. A total of 20% of *S. aureus* were classified as MRSA. Most isolates of *Streptococcus agalactiae* were susceptible to each of the antibiotics tested except for tetracyclin and cefoxitin, for which none were susceptible (Table 3). Most Gram- negative bacteria had low levels of susceptibility to quinolones (ciprofloxacin and levofloxacin) except for *Pseudomonas stutzei*. All bacteria showed higher levels of susceptibility to carbapenems (imipenem and meropenem), although levels of susceptibility were generally varied between the two. Some bacteria showed low levels of sensitivity to gentamicin, but susceptibility to amikasin was generally high; except for *Achromobacter xylosoxidans* and *Stenotrophomonas maltophilia*. Most bacteria had decreased sensitivity to cephalosporins (cefotaxime, ceftazidime, and cefepime) (Table 4).

**Table 3:** Antimicrobial susceptibility result for gram-positive bacteria July 2013–December 2015.

		**Antibiotic (% susceptible)**							
***Bacteria***	**Tested (*n*)**	**Ampicillin**	**Erytromycin**	**Tetracyclin**	**Chloramphenicol**	**Clindamycin**	**Linezolid**	**Vancomycin**	**Cefoxitin**
*Coagulase-negative staphylococci*	502	0.00	0.00	0.00	0.00	0.00	13.35	21.72	0.00
*Staphylococcus aureus*	169	0.00	76.41	48.82	74.05	90.05	98.54	95.58	69.33
*Viridans streptococci*	497	0.00	37.94	9.56	75.24	54.29	86.57	82.69	0.00
*Enterococcus faecalis*	71	56.25	17.87	14.70	59.15	0.00	92.36	94.08	0.00
*Bacillus sp*.	54	12.96	12.96	0.00	35.65	0.00	0.00	0.00	0.00
*Streptococcus agalactiae*	11	72.73	77.28	0.00	40.92	68.18	90.91	81.82	0.00

**Table 4:** Antimicrobial susceptibility result for gram-negative bacteria July 2013–December 2015.

		**Antibiotic (% susceptible)**									
**Bacteria**	**Tested (*n*)**	**Ciprofloxacin**	**Levofloxacint**	**Cefotaxime**	**Ceftazidime**	**Cefepime**	**Imipenem**	**Meropenem**	**Gentamicin**	**Amikasin**	**Cotrimoxazole**
*Eschericia coli*	785	23.24	29.06	37.88	39.09	50.02	91.58	94.57	62.87	89.59	28.30
*Klebsiella pneumoniae*	538	37.61	57.19	28.20	25.60	33.60	87.91	90.18	43.07	82.48	37.63
*Acinetobacter baumanii*	824	23.52	29.20	2.92	12.88	25.69	55.89	36.95	35.34	51.68	50.63
*Pseudomonas aeruginosa*	668	56.57	72.77	-	55.53	72.20	68.02	69.84	61.96	76.99	17.41
*Enterobacter cloacae*	103	63.56	73.19	35.37	38.59	60.37	89.18	84.98	59.53	95.11	56.28
*Proteus mirabilis*	106	59.66	61.55	74.79	80.33	87.43	44.50	87.74	68.72	89.56	50.46
*Stenotrophomonas maltophilia*	61	1.64	94.13	0.00	0.00	9.18	0.00	0.00	0.00	0.00	91.42
*Morganella morganii*	40	31.89	57.99	60.02	66.67	89.99	52.51	84.99	61.26	77.49	39.82
*Achromobacter xylosoxidans*	10	0.00	90.01	0.00	78.32	21.68	85.00	70.00	0.00	11.69	76.01
*Psedomonas stutzeri*	10	100.00	15.00	0.00	79.99	85.00	70.00	90.01	79.99	90.01	79.99
*Acinetobacter iwoffii*	25	51.97	84.01	57.31	48.40	61.31	81.60	70.54	71.00	71.00	70.67
*Pseudomonas putida*	16	27.06	25.00	0.00	50.00	43.75	58.31	27.75	49.99	93.75	7.81

## Discussion

Studies investigating the distribution of bacterial pathogens and their susceptibility in Indonesian hospitals are very few in number. The most similar study was conducted in Fatmawati Hospital, Jakarta at 2010, but the samples were recruited only from ICU patients (17). Another study reported the hospital acquired infection in two teaching hospitals in East Java, but did not describe the pathogen distribution nor resistance (13). Other studies have reported bacterial pathogens for diarrheal infections (18–20) and their resistance (21–23). Our study is able to add the distribution of bacteria and their susceptibility profile for patients across all ward types within an Indonesian tertiary hospital, and an area with unique population characteristics.

In our study, organisms were isolated from 63% of all cultures investigated, which is slightly lower than a study conducted in Jakarta Hospital ICU (64.7%) (17) and higher than a study conducted in General Hospital ICU in India (36.8%) (23). The most predominant organism isolated was *E. coli* which differs to Radji et al. who identified *Pseudomonas aeruginosa* (26.5%) as the most frequently isolated organism in Fatmawati Hospital (17). However, this difference is likely to reflect the study population investigated by Radji et al., which was restricted to ICU patients. In our study we also identified a predominance of Gram-Negative organisms in intensive care settings, including *A. baumannii* and *P. aeruginosa* and *K. pneumoniae*. Other studies in India found that *K. pneumoniae* and *E. coli* (24) were the most common organism isolated within ICU settings. The current study shows that—unlike reports in Malaysian studies—the bacteria routinely isolated from wounds were not dominated by *S. aureus* but a wider distribution was observed. Wong et al. reported *S. epidermidis* was isolated predominantly from acute wounds and *S. aureus* from chronic wounds (25).

As a definition for nosocomial infections the guidance by the Centers for Disease Control and Prevention's (CDC's) National Healthcare Safety Network (NHSN) was used, defining those as infections acquired in a hospital by a patient who was admitted for a reason other than that infection (26). We identified that most Gram-Negative organisms implicated in nosocomial infections were resistant to quinolones and cephalosporins, but remained susceptible to carbapenems and aminoglycosides (amikacin). This finding is similar to Kuntaman et al. assessing the susceptibility of Extended Spectrum Beta Lactamase (ESBL) producing bacteria to six antibiotics in 3 major cities in Indonesia (Surabaya, Semarang, and Malang) (27).

Hadi et al. also reported an increasing prevalence of ESBL (from 22 to 53%) and MRSA (from 18 to 24%) between 2010 and 2012 in Surabaya (9). Sheth et al. found that in India the sensitivity of Gram-negative organisms was better to ciprofloxacin (66.6–87.5%) and were 100% susceptible to imipenem, meropenem, and levofloxacin, yet was poorer to cefotaxime, ceftriaxone, ceftazidime, and cefoperazone. However, it should be noted that the study only looked at Intensive Care Unit patients, and not the general patient population within the tertiary hospital. In Madagascar, the antibiotic sensitivity of Gram-negative organisms was similar to the above, with 98.1–100% sensitivity to imipenem, 28–96.1% to ciprofloxacin, 24–100% to gentamicin, and 38–100% to cephalosphorine; with the exception of *A. baumannii*, which had lower sensitivity (56% susceptible) to carbapenems (28). In Singapore, bacterial susceptibility to third generation cephalosporins has decreased by 30% and the prevalence of ESBLs increased by 30.3% between 1990 and 2006. Furthermore, carbapenem resistance was estimated as endemic in Singapore (2), specifically for *A. baumannii* with up to 70% prevalence of resistance observed in tertiary hospital samples (29).

This report has certain limitations, firstly it has to be stated that it is difficult to distinguish infections from aseptic inflammation, especially in resource-restricted settings where patient presentations can be complex. Secondly, the culture results consistent with stain result and in conjunction with raised blood cell counts is not always definitive evidence of infection. However, we consider that these limitations do not apply to the majority of the patients seen and samples tested. Notwithstanding the above, this report has direct implications for local clinical management, and future healthcare planning. Existing guidelines for antibiotic treatment in Indonesian hospitals have been largely derived using data from developed countries which differ, often significantly, in terms of the causative bacteria profile. The implementation of such guidelines (through the AMR-control program) has been slow in Indonesia and generally restricted to tertiary hospitals initially, due to resources restrictions. Further plans to extend to all hospitals have now been enabled and the guidelines are reviewed with appropriate data sets on reported antimicrobial resistance, so that the guidelines are informed by the local context.

Our results highlight the limitations of transferring the evidence-base from other settings and provide a frame of reference for developing local guidelines to promote antimicrobial stewardship. The high rates of non-susceptibility to cephalosporins and quinolones that we identified in Sanglah Hospital may reflect their widespread and prolonged use for empirical therapy. A review of antibiotic guidelines is urgently needed both to accommodate the local pattern and susceptibility of bacteria causing infections and to improve the clinical knowledge of stopping and switching antibiotic prescriptions.

Early detection of bacterial resistance has the potential to directly impact on treatment success and reduce healthcare costs. Our study confirms a high burden of MDROs within Indonesia (21, 30, 31), which has important implications for healthcare planning and echoes results from a clinical audit of two government hospitals in East Java. At these sites 42% of all prescriptions for fever or surgical prophylaxis were deemed unnecessary, the choice of drug, dosage or duration of treatment of all antibiotic prescriptions were inappropriate for 15% and <1 quarter of prescriptions were in-line with best practice (32). One of the limitations of the current study is the potential risk of bias on the resistance prevalence when several antibiotic resistant isolates from the same patient were taken. This data was not readily available to the authors and as such could not be included in the current manuscript. However, such parameters, as well as the length of hospitalization, exact dates and dosages of drug administration and previous exposure to hospital treatment, would need to be included in prospectively collected data to increase the discriminatory power of future analyses. Continued surveillance of antimicrobial resistance is important both to monitor changes in the patterns that we have reported, and to enable benchmarking at other sites in Indonesia.

Our study reports the identification and distribution of pathogens in a major tertiary hospital of Indonesia for an under-reported yet very densely populated area of Southeast Asia in the context of global literature and provides a foundation for future local and national studies. Future studies are essential for mapping the spread and development of MDROs and for local and global strategies for effective control of infectious disease and drug resistance.

## Data Availability Statement

The datasets for this article are not publicly available because they have not been released yet to the public by the Indonesian Ministry of Health. Until that takes place requests to access the datasets should be directed to Dr Fatmawati, Head of the Microbiology Department at Sanglah Hospital, Denpasar, Bali. The request shall be sent via email to the following email address: info@sanglahhospitalbali.com.

## Ethics Statement

Ethical approval for this study was not required in accordance with national legislation and institutional requirements. Expressed permission from Sanglah Hospital was provided to the authors for the use of the anonymised, aggregated data, exclusively for the purposes of this study.

## Author Contributions

NB and WA conceptualized the idea of the study and designed the study. NF and NT collected and curated the data. DA and ZK interpreted the results and drafted the manuscript. All authors commented on the final version of the manuscript.

### Conflict of Interest

The authors declare that the research was conducted in the absence of any commercial or financial relationships that could be construed as a potential conflict of interest. Where authors are identified as personnel of the International Agency for Research on Cancer/WHO, the authors alone are responsible for the views expressed in this article and they do not necessarily represent the decisions, policy or views of the International Agency for Research on Cancer/WHO.
